# Factor-Reduced Human Induced Pluripotent Stem Cells Efficiently Differentiate into Neurons Independent of the Number of Reprogramming Factors

**DOI:** 10.1155/2016/4736159

**Published:** 2016-02-09

**Authors:** Andreas Hermann, Jeong Beom Kim, Sumitra Srimasorn, Holm Zaehres, Peter Reinhardt, Hans R. Schöler, Alexander Storch

**Affiliations:** ^1^Division for Neurodegenerative Diseases, Department of Neurology, Technische Universität Dresden, Dresden, Germany; ^2^Center for Regenerative Therapies Dresden (CRTD), Technische Universität Dresden, Dresden, Germany; ^3^German Center for Neurodegenerative Diseases (DZNE), Research Site Dresden, 01307 Dresden, Germany; ^4^Hans Schöler Stem Cell Research Center (HSSCRC), Max Planck Partner Group-MBL, School of Life Sciences, Ulsan National Institute of Science and Technology (UNIST), Ulsan, Republic of Korea; ^5^Department of Cell and Developmental Biology, Max Planck Institute for Molecular Biomedicine, Münster, Germany; ^6^Department of Neurology, University of Rostock, Rostock, Germany

## Abstract

Reprogramming of somatic cells into induced pluripotent stem cells (iPSCs) by overexpression of the transcription factors OCT4, SOX2, KLF4, and c-Myc holds great promise for the development of personalized cell replacement therapies. In an attempt to minimize the risk of chromosomal disruption and to simplify reprogramming, several studies demonstrated that a reduced set of reprogramming factors is sufficient to generate iPSC. We recently showed that a reduction of reprogramming factors in murine cells not only reduces reprogramming efficiency but also may worsen subsequent differentiation. To prove whether this is also true for human cells, we compared the efficiency of neuronal differentiation of iPSC generated from fetal human neural stem cells with either one (OCT4; hiPSC_1F-NSC_) or two (OCT4, KLF4; hiPSC_2F-NSC_) reprogramming factors with iPSC produced from human fibroblasts using three (hiPSC_3F-FIB_) or four reprogramming factors (hiPSC_4F-FIB_). After four weeks of coculture with PA6 stromal cells, neuronal differentiation of hiPSC_1F-NSC_ and hiPSC_2F-NSC_ was as efficient as iPSC_3F-FIB_ or iPSC_4F-FIB_. We conclude that a reduction of reprogramming factors in human cells does reduce reprogramming efficiency but does not alter subsequent differentiation into neural lineages. This is of importance for the development of future application of iPSC in cell replacement therapies.

## 1. Introduction

Reprogramming of somatic cells into induced pluripotent stem cells (iPSCs), which initially was achieved in mouse and human fibroblasts by ectopic expression of four transcription factors, OCT4 (also called OCT3/4 or Pou5f1), SOX2, KLF4, and c-Myc [1–6], is nowadays widely considered as a major breakthrough for regenerative medicine and has become a ground-breaking and competitive field of research during recent years. Their therapeutic potential was exemplarily shown in animal models of Parkinson's disease (PD) [7], sickle cell anemia [8], acute myocardial infarction [9], and diabetes [10], giving hope for future clinical applications in personalized cell repair strategies.

Originally, induction of pluripotency in somatic cells was achieved by forced expression of OCT4, SOX2, KLF4, and c-Myc via retroviral integration [1–6]. These procedures harbour some limitations for future clinical applications as reactivation or sustained expression of reprogramming transgenes may result in tumor formation [4], induce dysplasia [11, 12], and also impair the developmental potency of iPSC by altering the expression of transcription factors responsible for pluripotency [13, 14]. Additionally, remaining transgene expression may interfere with downstream differentiation experiments in vitro. Therefore, many strategies were used to generate iPSC without retroviral integrations including the use of nonintegrating adenoviruses [15], oriP/EBNA1-based episomal vectors [16], PiggyBac transposon systems [17, 18], transient transfection with reprogramming plasmids [19], Cre recombinase excisable viruses [20], and recombinant proteins [21, 22] and with synthetic RNA [23]. However, reprogramming with recombinant proteins is still of very low efficiency and, thus, needs further optimization before being available for routine use for the generation of iPSC for research and translational medicine.

Another strategy to minimize the risk for chromosomal disruption is to reduce the number of reprogramming factors. Indeed, generation of iPSC from mouse and human fibroblasts is possible without the use of c-Myc, although at a lower efficiency [24, 25]. Reprogramming with only OCT4 and KLF4 has been successfully achieved in adult mouse neural stem cells (NSCs) [26–28]. Furthermore, iPSCs were successfully generated from human fibroblasts using OCT4 and SOX2 [29] and from human NSC using OCT4 and KLF4 [30]. Finally, adult mouse and fetal human NSCs can also be directly reprogrammed to pluripotency by OCT4 alone [31, 32]. OCT4 alone seems to be able to induce pluripotency in primary somatic cells when combined with small molecules [33].

We recently demonstrated that neuronal differentiation is significantly less effective in one- and two-factor iPSC from murine adult NSCs suggesting limitations for the strategy of factor reduction to minimize the risk for chromosomal disruption as prerequisite for therapeutic use [34]. Since species differences may be responsible for this issue, we here investigate the neuronal differentiation behaviour of factor-reduced iPSC derived from human NSC. Our data could have important consequences for future clinical applications of factor-reduced iPSC, which will depend on an effective output of differentiated cells.

## 2. Methods

### 2.1. Derivation of iPSCs

For this comparative study, we used iPSCs derived from fetal human NSCs [32] and iPSCs produced from adult human fibroblasts [35]. iPSCs from fetal human NSCs were produced by retroviral introduction of either one (OCT4; iPSC_1F-NSC_) or two (OCT4, KLF4; iPSC_2F-NSC_) reprogramming factors [28, 31, 32]. iPSCs from adult human fibroblasts were generated by retroviral introduction of three (OCT4, KLF4, and SOX2; iPSC_3F-FIB_) or all four (OCT4, KLF4, SOX2, and c-Myc; iPSC_4F-FIB_) reprogramming factors as published in [35, 36]. iPSCs were cultured in colonies on feeder layers of mitotically inactivated mouse embryonic fibroblasts using standard procedures [32, 35].

### 2.2. Neuronal Differentiation of iPSCs

Neuronal differentiation of iPSCs was induced by coculture with PA6 stromal cells, which has been shown to promote neural induction by stromal cell-derived inducing activity (SDIA) and to generate high proportions of midbrain specific dopaminergic neurons from ESC and iPSC [34, 37]. Freshly passaged colonies were plated on confluent layers of PA6 cells, which had been propagated in *α*-Minimum Essential Medium (*α*MEM; Invitrogen) supplemented with 10% FCS (Sigma-Aldrich), 100 U/mL penicillin, and 100 *μ*g/mL streptomycin (Invitrogen) and had been seeded one day before on 0.1% gelatine-coated 4-well tissue culture plates (Nalge Nunc International, Rochester, NY, USA) with coverslips. After seeding, iPSCs were cultured on the PA6 cells in Glasgow Minimum Essential Medium (GMEM; Invitrogen) supplemented with 10% Knockout Serum Replacement (Invitrogen), 2 mM* L*-glutamine (Invitrogen), 1 mM sodium pyruvate (Invitrogen), 1x nonessential amino acids (Invitrogen), 0.1 mM *β*-mercaptoethanol (Sigma-Aldrich), 100 U/mL penicillin, and 100 *μ*g/mL streptomycin (Invitrogen). Medium was changed on day 4 and every other day following that. On day 14, medium was changed to DMEM supplemented with 1% N-2 (Invitrogen), 2 mM* L*-glutamine (Invitrogen), 1 mM sodium pyruvate (Invitrogen), 0.1 mM nonessential amino acids (Invitrogen), 0.1 mM 2-mercaptoethanol (Sigma-Aldrich), 100 U/mL penicillin, and 100 *μ*g/mL streptomycin (Invitrogen), and medium change was performed every second day thereafter. After 3-4 weeks, cells on coverslips were rinsed with phosphate-buffered saline (PBS; Invitrogen), fixed for 30 seconds with formalin-free fixative (Accustain; Sigma-Aldrich), and afterwards rinsed twice with PBS.

### 2.3. Immunocytochemistry after Neural Differentiation

Standard procedures were used [38]. In brief, fixed samples were incubated for 2 hours with blocking buffer consisting of PBS, 3% normal donkey serum (Jackson ImmunoResearch, Suffolk, UK), and 0.1% Triton X-100 (Serva, Heidelberg, Germany) and exposed to primary antibodies in blocking buffer overnight at 4°C. After incubation with primary antibodies, cells were rinsed four times with PBS and subsequently exposed to fluorescent-labeled Alexa Fluor (Invitrogen) secondary antibodies for 1 hour at room temperature (1 : 500). After rinsing in PBS, coverslips were counterstained with Hoechst 33342 (7.5 *μ*g/mL; Invitrogen), mounted onto slides in Vectashield mounting medium (Vector Laboratories, Burlingame, CA, USA), and sealed with nail polish. Microscopic analysis was performed using an inverted fluorescent microscope (DMIRE2, Leica Microsystems). On each coverslip, all colonies containing cells with a positive staining for a specific marker were counted and put into relation to the total number of colonies detected by Hoechst counterstaining. The following primary antibodies were used: rabbit anti-TUJ1 (1 : 2000; Covance; Princeton, NJ, USA), mouse anti-microtubule-associated protein 2 (anti-MAP2; 1 : 500; BD Pharmingen, Franklin Lakes, NJ, USA), rabbit anti-tyrosine hydroxylase (anti-TH; 1 : 500; Pel-Freez, Rogers, AR, USA), chicken anti-glial fibrillary acidic protein (anti-GFAP; 1 : 1000; Abcam, Cambridge, MA, USA), and mouse anti-galactosylceramidase (anti-GALC; 1 : 750; Millipore).

### 2.4. Statistical Analysis

Either two-sided unpaired *t*-test or one-way analysis of variance (ANOVA test) with Bonferroni adjusted* post hoc t*-test was performed to determine differences between different iPSCs. Results are provided as mean ± standard error of the mean (SEM).

## 3. Results

**Figure 1:**
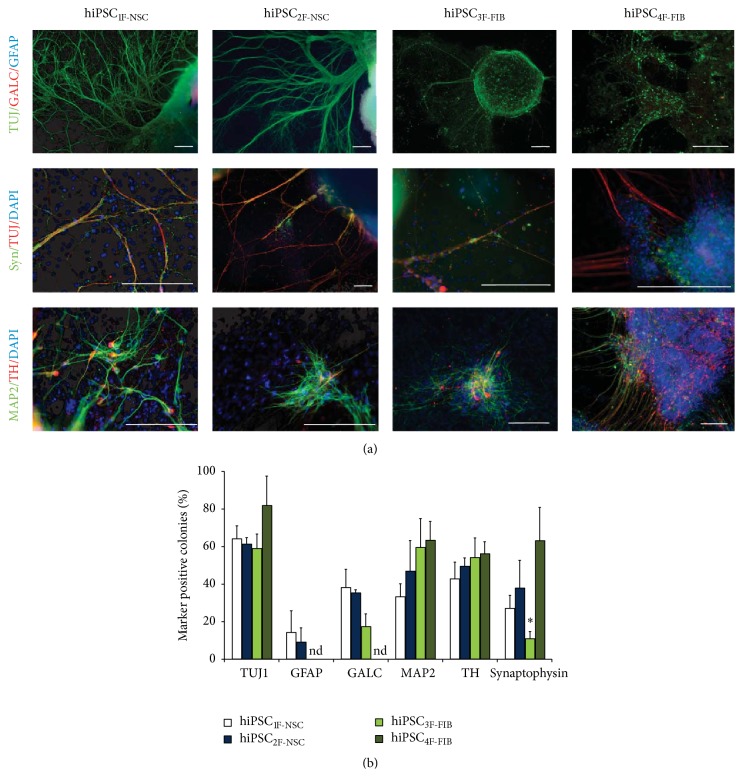
Comparison of neuroectodermal differentiation potential of human factor-reduced iPSCs. (a) Representative images of TUJ1^+^, GFAP^+^, GALC^+^, MAP2^+^, TH^+^, and Synaptophysin^+^ colonies generated by iPSCs derived from human fetal NSCs by one-factor reprogramming (OCT4, hiPSC_1F-NSC_), by iPSCs derived from human fetal NSCs by two-factor reprogramming (OCT4, KLF4; hiPSC_2F-NSC_), by iPSCs derived from human fibroblasts by three-factor reprogramming (OCT4, KLF4, and SOX2; hiPSC_3F-FIB_), and by iPSCs derived from human fibroblasts by classical four-factor reprogramming (OCT4, KLF4, SOX2, and c-Myc; hiPSC_4F-FIB_) after 3-4 weeks of differentiation on PA6 stromal cells. Scale bars represent 200 *μ*m. (b) Values are means ± SEM from at least three to four independent experiments. ^*∗*^ *p* < 0.05,* post hoc t*-tests with Bonferroni adjustment in comparison to hiPSC_4F-FIB_.

To assess fate determination towards neural lineages, colonies of different iPSC types were immunostained against the neuronal marker TUJ1, the astroglial marker GFAP, and the oligodendrocyte marker GALC after 3-4 weeks of differentiation. As expected, we found high numbers of TUJ1 colonies in hiPSC_4F-FIB_ (82 ± 16% of all colonies). Surprisingly, also factor-reduced hiPSC produced high amounts of neurons with similar percentages compared to hiPSC_4F-FIB_ (hiPSC_3F-FIB_  59 ± 8%, hiPSC_1F-NSC_  64 ± 7%, and hiPSC_2F-NSC_  61 ± 4%; *n* = 3-4; *F*-value: 2.916, *p* = 0.87; one-way ANOVA) (Figure 1(b)). No significant differences were observed in the quantification of the astroglial marker GFAP between hiPSC_1F-NSC_, 14 ± 11%, and hiPSC_2F-NSC_, 9 ± 7%; (*p* = 0.574, two-sided unpaired *t*-test; Figure 1(b)). For GALC^+^ oligodendrocytes we found significantly reduced numbers of positive colonies in hiPSC_3F-FIB_ compared to hiPSC_1F-NSC_ (*p* = 0.047,* post hoc* two-sided *t*-test with Bonferroni adjustment; one-way ANOVA; *F*-value: 5.557; *p* = 0.036) but not to hiPSC_2F-NSC_ (*p* = 0.114;* post hoc* Bonferroni *t*-test; hiPSC_1F-NSC_  38 ± 10%, hiPSC_2F-NSC_  35 ± 2%, and hiPSC_3F-FIB_  17 ± 7%).

Quantification of MAP2, a marker for mature neurons, revealed similar proportions of MAP2^+^ colonies in classical fibroblast-derived hiPSCs (hiPSC_4F-FIB_  63 ± 10% of all colonies) compared to factor-reduced fibroblast-derived hiPSCs (hiPSC_3F-FIB_  60 ± 17%) and NSC-derived hiPSCs (hiPSC_1F-NSC_  33 ± 7% and hiPSC_2F-NSC_   47 ± 16%); (*F*-value = 3.385, *p* = 0.062, one-way ANOVA). Synaptophysin was used as a marker for fully mature neurons indicating the presence of functional synapses. Factor reduction led to a significant reduction of Synaptophysin positive colonies in hiPSC_3F-FIB_ (11 ± 4%) compared to hiPSC_4F-FIB_ (63 ± 18%; *F*-value = 7.158, *p* = 0.009, one-way ANOVA; *p* = 0.01;* post hoc* Bonferroni *t*-test) but not when compared to factor-reduced NSC-derived hiPSCs (hiPSC_1F-NSC_  27 ± 7%, *p* = 0.078; and hiPSC_2F-NSC_  38 ± 15%, *p* = 0.363; both* post hoc* Bonferroni *t*-test).

Exemplarily, dopaminergic neuronal differentiation was investigated which would be of interest for Parkinson's disease. Colonies were quantified for presence of the marker protein tyrosine hydroxylase (TH) (Figure 1). 56 ± 6% TH-positive colonies were present after differentiation of classical fibroblast-derived hiPSC with no significant difference to factor-reduced fibroblast-derived hiPSC, hiPSC_3F-FIB_ (54 ± 11%), and to NSC-derived hiPSCs (hiPSC_1F-NSC_  43 ± 9% and hiPSC_2F-NSC_  50 ± 5%; *F*-value = 1.599, *p* = 0.251, one-way ANOVA; Figure 1(b)).

## 4. Discussion

An approach to minimize the risk for insertional mutagenesis and to simplify the reprogramming process in pluripotent stem cell research is the reduction of reprogramming factors [24–33]. Until recently, studies on factor-reduced human iPSC mainly focused on the derivation of individual iPSC clones without investigating their differentiation potential in detail. This, however, is of great importance when discussing possible clinical applications. The take-home message of the current study is that in contrast to murine factor-reduced iPSCs human NSC-derived factor-reduced iPSCs retain the full neuroectodermal differentiation potential. This is of major interest since this would allow factor reduction as an attempt to decrease the risk of malignancy after transplantation in humans. A combination of factor reduction and the use of protein reprogramming could be a significant step towards clinical application.

One major limitation of the factor reduction is the significantly decreased efficiency in iPSC generation. This also limits our study since only very few clones could be investigated. Nevertheless, these few clones showed a neuronal differentiation behaviour comparable not only to ESC and iPSC differentiation [34, 37] but also to mouse, rat, and human NSC differentiation [39–42].

The differences to our previous results of factor-reduced mouse iPSCs [34] could be versatile. Species to species differences are always possible. The factor-reduced mouse iPSCs were derived from postnatal NSCs, whereas the factor-reduced human iPSCs mentioned here were derived from fetal human NSCs. We cannot exclude the fact that this developmental difference could be a relevant factor influencing the postreprogramming differentiation capacity. Further studies like performing these experiments with factor-reduced iPSCs derived from adult human NSCs are warranted. Additionally, it would be interesting to investigate iPSCs derived from fetal human fibroblasts. Finally, one major difference is, however, the details of the proof of pluripotency in the different systems. In murine iPSC, generation of chimeras, germline transmission, and tetraploid embryo aggregation is the most compelling proof of pluripotency. These tests are obviously not possible with human iPSCs. Thus, one cannot fully rule out the fact that human iPSCs are not 100% reprogrammed and thus the different clones may vary more than in the murine system. Additionally, classical reprogramming could also lead to intermediates [43] as well as induced NSCs [43, 44]. This is, however, unlikely since all our iPSCs used here bear the differentiation potential into all germ layers [32, 35, 36]. A recent study by Hu et al. suggested that neural differentiation of iPSCs derived from human fibroblasts is less efficient and more variable across cell lines than in ESC [45]. This was shown to be independent from the technique used for reprogramming. This, however, makes it difficult to identify which contributors may have had an influence on the efficiency of neural differentiation in the individual iPSCs [45].

Another debate is about the protocol for neuronal differentiation we used within this study. We chose this protocol since it avoids an intermediate step of NSC propagation in which the potential well-behaving NSC subpopulations could overgrow not well differentiating ones, thereby masking potential differences in the neuroectodermal differentiation [46]. This is, however, on the cost of dealing with a coculture system with significant limitations concerning cell characterizations.

Nevertheless, since we herein prove the noninferiority of the neuroectodermal differentiation capacity of the human factor-reduced iPSCs and the differentiation capacity was well within the known range [37], we believe that human factor-reduced iPSCs behave similar to classical iPSCs and thus might be more suitable for clinical application in the future.

## 5. Conclusion

We herein show that, in contrast to murine factor-reduced iPSCs, human NSC-derived factor-reduced iPSCs retain the full neuroectodermal differentiation potential. This is of major interest since this would allow factor reduction as an attempt to decrease the risk for malignancy after transplantation in humans. A combination of factor reduction and the use of protein reprogramming could be a significant step towards clinical application.
